# Glucose-Induced Transcriptional Hysteresis: Role in Obesity, Metabolic Memory, Diabetes, and Aging

**DOI:** 10.3389/fendo.2018.00232

**Published:** 2018-05-28

**Authors:** Charles V. Mobbs

**Affiliations:** Icahn School of Medicine at Mount Sinai, New York, NY, United States

**Keywords:** glucose, transcriptional hysteresis, metabolic memory, obesity, diabetes, aging

## Abstract

During differentiation transient, inducers produce permanent changes in gene expression. A similar phenomenon, transcriptional hysteresis, produced by transient or prolonged exposure to glucose, leads to cumulative, persistent, and largely irreversible effects on glucose-regulated gene expression, and may drive key aspects of metabolic memory, obesity, diabetes, and aging, and explain the protective effects of dietary restriction during aging. The most relevant effects of glucose-induced transcriptional hysteresis are the persistent effects of elevated glucose on genes that control glucose metabolism itself. A key observation is that, as with the lac operon, glucose induces genes that promote glycolysis and inhibits gene expression of alternative metabolic pathways including the pentose pathway, beta oxidation, and the TCA cycle. A similar pattern of metabolic gene expression is observed during aging, suggesting that cumulative exposure to glucose during aging produces this metabolic shift. Conversely, dietary restriction, which increases lifespan and delays age-related impairments, produces the opposite metabolic profile, leading to a shift away from glycolysis and toward the use of alternative substrates, including lipid and ketone metabolisms. The effect of glucose on gene expression leads to a positive feedback loop that leads to metastable persistent expression of genes that promote glycolysis and inhibit alternative pathways, a phenomenon first observed in the regulation of the lac operon. On the other hand, this pattern of gene expression can also be inhibited by activation of peroxisome proliferator activating receptor transcription factors that promote beta oxidation and inhibit metabolism of glucose-derived carbon bonds in the TCA cycle. Several pathological consequences may arise from glucose-induced transcriptional hysteresis. First, elevated glucose induces glycolytic genes in pancreatic beta cells, which induces a semi-stable persistent increase in insulin secretion, which could drive obesity and insulin resistance, and also due to glucose toxicity could eventually lead to beta-cell decompensation and diabetes. Diabetic complications persist even after complete normalization of glucose, a phenomenon known as metabolic memory. This too can be explained by persistent bistable expression of glucose-induced glycolytic genes.

## Introduction

It is a truth universally acknowledged that during differentiation transient exposure to an inducer produces permanent change in gene expression. A classic example is the induction of the Glass gene during differentiation of *Drosophila* photoreceptor cells, which is induced by transient exposure to a differentiation factor. Subsequently, the product of the Glass gene induces photoreceptor-specific genes as well as itself perpetuating the differentiated state (1).

Persistent effects depending on the history of the system may be referred to as “hysteresis.” The term hysteresis is borrowed from physics and engineering, meaning “dependence of the value of a property on the past history of a system” (The New Shorter Oxford Dictionary) and is now used to describe a wide range of biological phenomena. An example of mechanical hysteresis is that after repetitively bending a wire, the wire eventually breaks, thus exhibiting a property of, metaphorically, “remembering” the number of times it has been bent. A more refined version of hysteresis exhibits so-called bistability in which the state of the system is reversible, but the state of the system depends on the history of the system. A classic example of bistability in a hysteretic system is the Schmidt trigger switch used in electrical engineering. If the switch is off and the voltage increases from 0 to 5 mV, for example, the switch is not activated until reaching 5 mV. However, if the trigger begins in the activated position at 5 mV and voltage decreases from 5 to 0 mV, the switch does not turn off until the voltage reaches 0 mV. Therefore, at 3 mV, the trigger will be either on or off, depending on the history of the circuit. As described below, this bistable behavior is critical for prospects of reversing pathologies caused by transcriptional hysteresis.

Other examples of persistent effects on gene expression include those produced by estrogen (2, 3). These effects plausibly lead to age-related impairments in female reproductive function due to cumulative and persistent effects of estradiol on neuroendocrine function (4–6).

## Transcriptional Hysteresis in Glucose-Regulated Genes

The purpose of our initial studies was to develop a model for mechanisms driving aging in general and in particular to elucidate mechanisms by which dietary restriction (DR) broadly increases lifespan and delays age-related impairments [reviewed in Ref. (7)]. Based on the transcriptional hysteresis of estradiol-regulated genes, it was hypothesized that a similar phenomenon might drive the process of aging (6, 8). Initially, it was not obvious how transcriptional hysteresis would drive the process of aging or mechanisms of DR since in contrast to hysteresis in estrogen-regulated genes that drive age-related reproductive impairment, the relevant molecule in aging was not obvious. It was, therefore, hypothesized that diabetes might serve as a similar model, since in this case the relevant molecule, glucose, was widely accepted as driving pathology. One motivation for this hypothesis was that diabetic complications appear to be driven by cumulative, progressive, persistent, and largely irreversible effects of elevated glucose even after almost complete normalization by islet transplantation (9), similar to the cumulative and (largely) irreversible effects of estradiol (6, 8). Support for this hypothesis as a mechanism for mediating the protective effects of DR was that DR decreases blood glucose and total exposure to blood glucose as was indicated by reduced levels of hemoglobin A1c (10), a marker for cumulative glucose exposure over the previous 90 days widely used as an indicator of glucose control in patients with diabetes. This was interpreted as support for the hypothesis that glucose drives the aging process by non-enzymatic glycation of proteins (which drives the increase in hemoglobin A1c) (11). Indeed, Cerami coined the term for such glycated proteins advanced glycated endproducts to perhaps emphasize this hypothesis.

These observations supported a role for glucose driving the aging process, although our analysis of transcriptional hysteresis suggested an alternative mechanism to non-enzymatic glycation (6, 8). Subsequent results of the DCCT trials supported the clinical relevance of persistent effects of elevated glucose in patients with Type 1 diabetes (12). This landmark paper had many implications. First, it clearly demonstrated the major clinical benefit of reducing blood glucose using intensive insulin therapy, which involves significantly greater resources from both health care professionals and patients than standard therapy, and thus, significantly supports the hypothesis that it is indeed elevated blood glucose that drives diabetic complications. However, an unexpected result was that even after patients reverted to standard therapy (such intensive insulin therapy is extremely difficult to maintain over several years), complications were still delayed. This observation gave rise to the concept of “metabolic memory” (13), remarkably consistent with the proposal that diabetic complications are driven by cumulative largely irreversible effects of glucose on gene expression (6, 8).

Since metabolic memory appears to drive diabetic complications, and in particular the apparent irreversibility of metabolic memory is perhaps the major challenge in treating diabetic complications, the mechanisms mediating this phenomenon have been of great interest in the field of diabetes. Several elegant studies have demonstrated persistent epigenetic effects on gene expression after transient elevation of elevated glucose. An early example of such a persistent effect of elevated glucose was the report by Roy et al., who demonstrated that a transient increase in glucose produced an increase in expression of fibronectin in human endothelial cells that persisted even after several passages *in vitro* (14). Similarly, Kowluru et al. demonstrated that hyperglycemia in Type 1 diabetic rats produced oxidative stress that was not reversed by almost complete normalization of blood glucose (15). Consistent with these results, El-Osta et al. made the interesting observation that while hemoglobin A1c generally predicts the development of diabetic complications, less than 25% of the variance of complications can be explained by this parameter (16). They, therefore, proposed that random spikes of glucose could produce persistent effects on gene expression relevant to diabetic complications. Based on this hypothesis, they went on to demonstrate that a transient increase in glucose produced persistent elevation of the pro-inflammatory gene p65, a subunit of NF-KappaB, in endothelial cells both *in vitro* and *in vivo*, the latter of which persisted at least 6 days after restoration of normoglycemia (16). A similar phenomenon was observed in the expression of monocyte chemoattractant protein 1 and vascular cell adhesion molecule 1, both of which are induced by NF-KappaB (16).

While these were indeed landmark studies, the physiological significance of these markers for transcriptional hysteresis of glucose-regulated genes was not entirely evident. However, it was observed that DR produced a robust profile of gene expression regulating metabolism, entailing a restructuring of metabolic pathways to reduce glucose metabolism (17) and promote the use of alternative substrates, including beta oxidation and ketone metabolism (18–20), associated with enhanced protein turnover (21), increased activity of the pentose pathway (18, 22), and increased mitochondrial respiration (20, 23). Conversely, on the basis of a large-scale analysis of gene expression, Lee et al. concluded that “aging was associated with transcriptional alterations consistent with a metabolic shift from fatty acid to carbohydrate metabolism” and that dietary restriction “resulted in alterations in gene expression consistent with preserved fatty acid metabolism” through “transcriptional reprogramming” (18).

Since plasma glucose is reduced by DR, this led to the hypothesis that reduction in blood glucose leads to the pattern of metabolic gene expression observed (6, 8). It was not, however, until re-assessment of the results of long-term effects of estrogen that estrogen persistently induced estrogen-induced parameters, including gene expression, and persistently inhibited estrogen-inhibited parameters, that the hypothesis was developed that chronic exposure to glucose produces similar persistent effects (that is, chronic glucose persistently induces glucose-induced genes and persistently inhibits glucose-inhibited genes). In turn, it was proposed that diabetes accelerates this process (6, 8).

## Glucose Induces Genes Promoting its Own Metabolism: Potential Role in Obesity and Diabetes

Thus, it had been established that DR produces the metabolic switch discussed above (away from glycolysis and toward alternative substrates etc.) that aging produces the opposite profile and that glucose, like estrogen, could produce persistent effects on gene expression. However, the persistent effects of neither estrogen nor glucose provided a satisfactory explanation for how these persistent effects drive age-related impairments and, in the case of glucose, diabetic complications. The solution to this conundrum was suggested by considering the lac operon.

A general principle of metabolic regulation, suggested by the discovery of the Pasteur effect in yeast, is that substrates induce the machinery for their own metabolism. For example, yeast adapt to environmental conditions using a similar metabolic logic as mammals, such that when glucose is highly available (for example, as extracted from grape juice) it is used as the main fuel to produce ATP, and excess carbons are excreted in the form of ethanol to be used as fuel when glucose is less available (therefore, ethanol serves a similar function as that served by stored lipids in animals). Thus, depletion of glucose inhibits glycolysis and activates pathways for metabolism of ethanol (24). This principle became canonical with studies of the lac operon in bacteria, in which lactose induces both the activity and the gene expression of beta-galactosidase, the rate-limiting enzyme for the metabolism of lactose (25). A perhaps underappreciated aspect of the lac operon, however, is that glucose suppresses the operon even in the presence of lactose (24). Similarly, glucose inhibits alternative metabolic pathways in yeast (24, 26) and fungi (27). In most organisms and cell types, glucose is generally the preferred metabolite to produce ATP, possibly because glucose is the ultimate source of almost all energies in the biosphere (with the possible exception of tube worms, which apparently use hydrogen sulfide produced by thermophilic bacteria as the main source of energy) since glucose is the ultimate product of photosynthesis. However, similar to yeast and mammals, most organisms exhibit adaptive responses when glucose is depleted.

Mammals exhibit similar metabolic logic. Thus, in mammalian pancreatic (INS-1) beta-cells, glucose induces several genes coding for key glycolytic enzymes, including phosphofructokinase (PFK), glyceraldehyde phosphate dehydrogenase, and pyruvate kinase (28). The glucose analog 2-deoxyglucose did not mimic these effects, supporting that the induction of these genes is mediated by glucose metabolism (28). Interestingly after exposure to elevated glucose, these cells exhibit a form of physiological hysteresis, in that even after returning to relatively low levels of glucose insulin secretion continued to be elevated, suggesting a persistent effect of glucose on insulin secretion (28). Since insulin secretion is driven by glucose metabolism (29), this represents one of the first examples of glucose-regulated gene expression driving a physiological form of hysteresis. Although at this point, actual hysteresis of glucose-regulated metabolic genes had not yet been established, it would have been a plausible hypothesis since apparently glucose metabolism drove expression of glucose-metabolizing genes, which would be predicted to produce a metastable positive feedback loop (see below).

It should be noted that this pancreatic form of hysteresis could plausibly drive both age-related obesity and Type 2 diabetes. Insulin secretion increases with age, which is generally attributed as a compensation for age-related insulin resistance (30). However, there is no plausible explanation for why insulin resistance should increase with age. It is, therefore, just as plausible that gradual elevation of insulin secretion due to hysteretic effects of glucose on beta-cell function causes age-related insulin resistance (31). Type 2 diabetes does not occur until pancreatic decompensation, likely caused by glucose toxicity (31) through mechanisms described below. Similarly, increased insulin promotes storage of lipids in adipose tissue; so again a primary hysteretic drive increasing insulin secretion could plausibly be the cause of obesity as well (31). Thus, this simple mechanism could economically explain the otherwise somewhat mysterious relationship between Type 2 diabetes and obesity. Conversely, this mechanism could explain the otherwise somewhat mysterious effect of DR to increase insulin sensitivity (22). Furthermore, it is generally assumed that because weight loss in obesity improves glucose homeostasis and can even reverse diabetes, this implies that obesity causes diabetes. However, weight loss is almost always produced by reducing caloric intake, which in turn reduces glucose levels, which in turn reduces insulin secretion. Whether the hysteresis in beta cells is actually reversed by weight loss is an open question (a question addressed below), but the fact that weight loss is so difficult to maintain suggests that indeed the hysteresis is not reversed, but the effects are only reduced by fairly extreme caloric intake. As described below, there is a certain probabilistic component to transcriptional hysteresis, so it may be that the difference between obese and diabetic individuals and their thinner counterparts could be largely a matter of chance. This possibility should encourage those inclined to blame the victim to reconsider.

Another interesting example of glucose-regulated gene expression increasing glucose metabolism is the inhibition of pyruvate dehydrogenase kinase (PDK) by glucose in cultured human muscle cells (32). PDK phosphorylates pyruvate dehydrogenase (PD), thereby inhibiting its activity and thus inhibiting the transfer of pyruvate to the mitochondria, the main source of ATP production from glucose-derived carbons. This provides a major regulatory mechanism to reduce the use of glucose-derived carbons to make ATP, in the context, for example, of switching to lipid oxidation when glucose levels drop (e.g., a well-established phenomenon in the whole body during fasting or even sleeping). The major PDK isoform implicated in a wide range of such metabolic switching is PDK4. In cultured human muscle cells, reduction of glucose induces PDK4, an effect reversed by insulin, suggesting that the inhibition of PDK4 by glucose is driven by glucose metabolism (32). As with glycolytic gene expression, the inhibition of PDK4 by glucose would be expected to induce glucose metabolism; to the extent that the signal for glucose-regulated gene expression is produced by glucose metabolism itself, this would also suggest a self-perpetuating positive feedback semi-stable state promoting glucose-regulated gene expression. As indicated above, mammals optimize metabolic processes generally by metabolizing glucose when blood glucose is plentiful. This occurs during the primary time of eating: day for diurnal animals, night for nocturnal animals. When glucose is less abundant, due to sleep or prolonged fasting, there is a switch over to lipid metabolism (beta oxidation) and to a lesser extent ketone metabolism (principally in the brain). This switch occurs in the whole body, easily measured by indirect calorimetry. Free fatty acids (FFAs) become available during prolonged fasting when blood glucose levels fall, thus reducing insulin secretion, which leads to release of FFAs into the blood. As with most metabolites, FFAs induce their own metabolism, for example, in the liver and other tissues by induction of the transcription factor peroxisome proliferator activating receptor (PPAR) alpha subtype (PPAR-alpha). When activated by FFAs, PPAR-alpha induces metabolic enzymes that promote metabolism of FFAs, particularly carnitine palmitoyltransferase isoform 1a (CPT1a), the rate-limiting enzyme for lipid metabolism (beta oxidation). It is, therefore, of particular interest that pharmacological activation of PPAR-alpha by fenofibrate, an FDA-approved drug to treat dyslipidemia (generally, elevated blood lipids) specifically upregulates PDK4, which would effectively reduce glucose metabolism (32). Thus, glucose and FFAs are in transcriptional tension, which, as indicated above, would be expected to result in oscillating semi-stable transcriptional states. Demonstration that lipid and ketone metabolisms reduces glucose metabolism motivated the proposal that elevated FFAs in the blood leads to increased lipid metabolism and therefore, reduced glucose metabolism (33). In the context of fasting, this is an adaptive response since FFAs are generally only elevated during fasting, when glucose levels are limiting. The increase in FFA oxidation in peripheral organs (e.g., liver and muscle) spares glucose for the brain. However, in the context of obesity caused by nutritional over-abundance, this phenomenon becomes maladaptive, promoting insulin resistance and ultimately diabetes.

## Glucose Hysteresis of Glucose-Regulated Genes: Return to the lac Operon

The studies described above demonstrated that glucose produces a gene expression profile that enhances glucose metabolism. Since glucose metabolism in pancreatic beta cells drives insulin secretion, persistent glucose-induced metabolism in the beta cell would be expected to drive associated persistently elevated insulin secretion, leading to obesity and, following insulin resistance and beta cell “burn-out,” Type 2 diabetes. Those studies also provided a potential mechanism for transcriptional hysteresis by the way of potentially bistable positive feedback loops. However, these studies did not directly demonstrate transcriptional hysteresis in glucose-regulated genes.

A major precedent for such a mechanism was again provided by studies in the lac operon (34, 35). The study by Ozbudak et al. (34), carried out entirely in physics departments, provided a major breakthrough in the understanding of transcriptional hysteresis. The study was mathematically elegant and addressed many aspects of transcriptional hysteresis, so is well worth studying in detail. However, three main findings from these studies are particularly relevant to analysis of transcriptional hysteresis in glucose-regulated genes. These studies demonstrated that the lac operon exhibits transcriptional hysteresis, but also revealed that mechanistic details are not previously observed in the few previous known examples of transcriptional hysteresis. A dose–response analysis of inducer (TMG) vs. induction of the lac operon (using a reporter) at the single-cell level demonstrated that there is a bistable state of induction of the lac operon in individual cells. Thus, the operon was either on or off. This demonstrated that the observed dose–response curve is not due to gradual induction of gene expression, but rather to the number of cells induced at each dose: the higher the dose, the greater the number of cells induced. The key observation of the study, however, was that the number of cells induced at a given dose of TMG depends on the history of the system. Thus, when the concentration of TMG was gradually increased from about 3 μM (at which dose the operon was inhibited in essentially all cells) to about 30 μM (at which dose the operon was active in essentially all cells), the operon only became active in an appreciable number of cells at about 15 μM, and maximally active at the maximum concentration of TMG, 30 μM. In contrast, when the concentration of the TMG was gradually decreased from a concentration of 30 μM down to 3 μM, the operon stayed active in essentially all cells until about 5 μM. Thus, when gradually increasing from the lowest dose, the operon was only active in a small minority of cells between 6 and 18 μM. In contrast, when gradually decreasing from the highest dose, the operon was active in essentially all cells at 6–18 μM, but reversed to non-activity at 5 μM. The most important therapeutic implication was that apparently irreversible effects, as observed in metabolic memory associated with diabetes, may in principle be reversible if the effective substrate availability is sufficiently reduced. Thus, the lac operon exhibits classic bistable hysteresis, essentially the same as the Schmidt trigger. Finally, this study clearly demonstrated the key phenomenon supporting this bistable hysteretic state: a semi-stable self-sustaining positive feedback loop, in which TMG induces the lactose transporter LacY (which also transports TMB), which then promotes further TMG uptake, which further induces LacY, etc. This kind of positive feedback loop has been proposed to be constituted of a fundamental feature of bistable transcriptional hysteresis (36).

This landmark study had three major implications for the existence of transcriptional hysteresis in glucose-regulated genes. First, the lac operon is the classic example of a metabolic gene inducing its own metabolism and demonstrating that it exhibits transcriptional hysteresis that strengthened the likelihood that glucose-regulated genes regulating glucose metabolism would also exhibit hysteresis (not yet demonstrated at that time). Second, and of great potential therapeutic significance, the transcriptional hysteresis of the lac operon is bistable, implying reversibility, a phenomenon not previously observed for the examples of transcriptional hysteresis described above. Finally, the demonstration of a positive feedback transcriptional mechanism supported the hypothesis described above that such a positive feedback mechanism could explain transcriptional hysteresis of glucose-regulated genes.

A final theoretical implication of these studies is how metabolite-induced transcriptional hysteresis might be adaptive. A bistable state of transcriptional activity prevents premature reversal of the transcriptional state. Thus, the presence of lactose predicts that lactose is most likely to be present in the future, and a reversal of the lac operon would prematurely impair the ability to metabolize lactose and would be maladaptive if, as is likely in this context, lactose is available again. The same logic applies to glucose-induced transcriptional hysteresis and to hysteresis of nutrient-sensing hypothalamic neurons as described below.

## Reversing Glucose-Induced Transcriptional Hysteresis: Implications for Metabolic Memory in Diabetic Complications and Aging

The potential reversibility of transcriptional hysteresis in the lac operon, but only at very low levels of inducer, suggested that the apparent irreversibility of diabetic complications, even after complete normalization of blood glucose, is due to normal glucose levels not being low enough. In the course of studies of the basic mechanisms of how glucose regulates hypothalamic gene expression, it was discovered that the ketone 3-hydroxybutyrate (3-OHB), which is metabolized to ATP, did not mimic effects of glucose, but rather blocked them (37). This study was carried out to assess the role of AMP/ATP ratios in mediating glucose-regulated hypothalamic gene, clearly demonstrating that these AMP/ATP ratios do not mediate these effects of glucose. Instead, these studies supported our previous studies implicating NADH as the key metabolite (38).

This led us to assess if elevated ketones might reverse diabetic complications (which does not occur even with complete normalization by blood glucose) by a combination of reducing glucose levels and reducing glucose metabolism (39). Blood 3-OHB levels were increased chronically using a ketogenic diet, which were being studied based on its remarkable ability to reverse obesity (40). Nephropathy was used as a model of diabetic complications in mouse models of both Type 1 and Type 2 diabetes (39). After diabetic nephropathy developed (as indicated by urinary albumin/creatinine ratios), mice were then on the ketogenic diet; controls were maintained on a chow diet. Within a few weeks, the diabetic nephropathy was completely reversed, as was the expression of kidney genes differentially expressed in the diabetic mice. This demonstrated proof of principle that metabolic memory can in fact be reversed. It was further demonstrated that 3-OHB is directly protective against oxidative stress. The efficacy of the ketogenic diet to reverse other diabetic complications and related age-related impairments, as well as some forms of cancer, are currently being assessed. Since DR also produces high levels of 3-OHB, it is possible that this is how DR increases lifespan and delays age-related diseases.

Nevertheless until that time transcriptional hysteresis had not been demonstrated in glucose-regulated genes that themselves regulate glucose metabolism. To assess this phenomenon directly, effects of glucose concentration on genes that regulate glucose metabolism, and reversibility of these effects, were assessed (41). These studies were carried out *in vitro*, examining effects of glucose on gene expression in a Schwann cell line, a cell type chosen for its relevance to diabetic neuropathy (42), but amenable to culturing *in vitro* (43). Elevated glucose induced almost all genes that stimulate glycolysis, inhibited genes that block glycolysis, and inhibited genes that promote metabolic pathways that are alternative to glycolysis (the pentose pathway, fatty acid oxidation pathway, and even the TCA cycle) (41). Remarkably, however, these effects were not observed even after 7 days of exposure to elevated glucose. However, since diabetic complications develop only slowly over weeks in mice and over years in humans, the Schwann cell line was maintained at elevated or controlled glucose levels for 8 weeks, then gene expression was examined. The pattern of metabolic gene expression indicated above (glucose-induced glycolysis and inhibition of lipid metabolism) was at the 8-week time point. Furthermore, when the cells previously exposed to elevated glucose were returned to normal glucose for 7 days, the observed profile of gene expression was not reversed. This was the first clear example of transcriptional hysteresis induced by glucose on metabolic genes that regulate glucose (41).

The functional significance of these changes in gene expression was demonstrated by direct measurement of glycolysis, which was elevated when the gene expression profile above was observed, and measure of reactive oxygen species, which were elevated when glycolysis was elevated. Using other metabolic substrates to probe likely metabolites mediating the effects on reactive oxygen species led to evidence that the key metabolite was NADH, rather than ATP (41).

The results of metabolite analysis also supported the hypothesis that NADH perpetuates transcriptional hysteresis in glucose-regulated genes. Further molecular analysis using chromatin immunoprecipitation assays indicated that glucose-induced transcriptional hysteresis is associated with persistently reduced chromatin binding of PPAR-gamma to genes promoting beta oxidation or otherwise inhibiting glycolysis, with no change in over all PPAR-gamma protein (41). These results are consistent with studies described above in which pharmacological activation of PPAR activity inhibits glucose-induced gene expression (32). Furthermore, pharmacological activation of both PPAR-alpha and PPAR-gamma prevented, but did not reverse, glucose-induced transcriptional hysteresis. Finally, glucose-induced transcriptional hysteresis was associated with persistent changes in glucose-regulated metabolic genes, although the direction of change in DNA methylation did not always correlate with persistent changes in gene expression. In short, this study demonstrated for the first time glucose-induced transcriptional hysteresis in genes regulating glucose metabolism, and the pattern of these persistent effects is consistent with observations in the lac operon suggesting that the hysteresis is driven by a positive feedback loop.

Another intriguing mechanism that might drive hysteresis of glucose-regulated genes entails the transcription factor HMGA1. Defects in this transcription factor reduce expression of the insulin receptor, thus inhibiting glucose metabolism (44). On the other hand, glucose induces activity of this transcription factor (45). Therefore, a plausible mechanism of hysteresis in glucose-regulated gene expression is that glucose induces HMGA1 activity, which induces the insulin receptor, promotes glucose metabolism, and further stimulates HMGA1 activity. This would produce a positive feedback loop which would be expected to produce persistent expression of glucose-induced gene expression.

## Mechanisms of Glucose Toxicity in Diabetic Complications and Aging

Since elevated glucose persistently drives expression of genes that preferentially stimulate glucose metabolism and decreases alternative metabolic pathways, whereas DR produces the opposite metabolic pattern, it is of interest to understand why this transcriptional hysteresis of glucose-regulated metabolic genes would be relatively more toxic whereas the opposite profile would be relatively protective. One obvious toxic effect is the inhibition of the pentose pathway, which is the main source of NADPH in the cytoplasm and thus the main source of anti-oxidant defenses. As to the relative toxicity of why glycolysis would be more toxic than beta oxidation, it was noted that when cells produce ATP from glycolysis, the main source of energy that ultimately drives ATP synthase entails reduction of NADH at ETC complex 1, which is a major source of reactive oxygen species (46–48), whereas the main source of energy from beta oxidation is reduction of FADH2 at ETC complex 2, which produces far fewer reactive oxygen species (49, 50). This hypothesis is supported by high-throughput RNAi screens, which have demonstrated that inhibition of complex 1 increases lifespan, whereas inhibition of complex 2 decreases lifespan (51, 52).

## Role of the Hypothalamus in Mediating Whole-Body Cumulative Toxic Effects of Glucose *via* Glucose-Induced Transcriptional Hysteresis

While glucose-induced transcriptional hysteresis appears to be cell autonomous, there is another layer of regulatory complexity that should be considered. Neurons in the ventromedial hypothalamus are uniquely specialized to sense and respond to nutritional state, and thereby, regulate energy balance and glucose homeostasis (53). Indeed, hypothalamic neurons sense glucose by a highly similar mechanism as pancreatic beta cells, entailing expression of the pancreatic form of glucokinase (38, 54). Glucose-sensing neurons in the hypothalamus play a major role in regulating whole-body glucose homeostasis. For example, interrupting glucose metabolism in these neurons produces immediate counterregulatory responses that mimic those produced by hypoglycemia (55). Many studies have demonstrated that hypothalamic nutrient-sensing neurons regulate peripheral glucose metabolism and even pancreatic beta-cell secretion (53).

Fasting (56) and hypoglycemia (57) produce similar responses as dietary restriction produces in many other tissues (including, induction of PDK4 and CPT1a), clearly indicating a shift away from glycolysis toward beta oxidation. The induction of genes that promote beta oxidation was initially surprising because it has generally been assumed that the brain does not utilize lipid oxidation to produce ATP. Rather, it is generally thought that during conditions of low glucose the brain preferentially metabolizes ketones, which in fact reduce brain metabolism of glucose (58) in a classic example of substrate competition similar to that between glycolysis and beta oxidation (33). Nevertheless, the genes promoting beta oxidation were expressed both in the hypothalamus and in the cortex (56). However, in general, fasting only induced genes promoting beta oxidation (e.g., CPT1a) and genes inhibiting glucose metabolism (e.g., PDK4), and inhibited genes promoting glucose utilization (e.g., PFK) in the hypothalamus, not the cortex (56).

Dietary and fasting of course entail changes in many factors that could plausibly mediate the effects of fasting on hypothalamic gene expression. For example, much evidence supports that effects of fasting on hypothalamic gene expression (e.g., NPY) and on functions regulated by the hypothalamus (e.g., reproductive function) are mediated by reduction in the hormone leptin (59). This hormone is produced in proportion to adiposity and, therefore, serves as an indicator to the hypothalamus, which expresses leptin receptors, the level of adipose stores, which of course decrease during fasting and DR (60). However, we demonstrated that the profile of metabolic gene expression produced by fasting in the hypothalamus (again, the same profile as produced by DR in peripheral tissues) is mediated by glucose, not leptin. First, in contrast to hypothalamic neuropeptide gene expression (e.g., increased AgRP and decreased POMC) which is similar in fasting and leptin-deficient mice (61), only fasting, not leptin deficiency, produces the profile of metabolic gene expression as DR (56). Similarly, taking hypothalamic slices from fasted mice, we observed that glucose, but not leptin, reversed this profile of expression within 4 h (56). Furthermore, insulin-induced hypoglycemia rapidly (within 4 h) produced the same metabolic profile (57)—leptin does not change that quickly and there was no change in adiposity in that short period of time.

Many lines of evidence have suggested that hypothalamic neurons play a key role in regulating lifespan (62) and age-related diseases such as obesity and diabetes (53), and even cancer (63). Of particular importance, reducing hypothalamic inflammation increases lifespan (62). In this context, it is particularly pertinent that enhanced inflammation in microglia, effectively the immune cells of the brain, produces exactly the same profile of metabolic genes as glucose-induced hysteresis (64). Consistent with these observations, effects of DR to increase lifespan are mediated by the ASI nutrient-sensing neurons in *Caenorhabditis elegans*, arguably the *C. elegans* equivalent of mammalian nutrient-sensing hypothalamic neurons (65). Furthermore, hypothalamic neurons controlling energy balance exhibit bistable hysteresis (66). In this case, the hysteresis observed was due to fasting. After fasting, there is a bistable state produced by the orexigenic hormone ghrelin, which even after reversal tends to maintain itself and resists reversal by anorectic stimuli hours after ghrelin is removed (66). This can be rationalized as an adaptive response, in that it is adaptive to produce a greater threshold of satiety after prolonged fasting, to promote more robust re-feeding and restoration of metabolic stores after prolonged fasting, an indication of environmental nutritional deficit, compared to the context of relative satiety (in which most humans find themselves). It is, therefore, hypothesized that glucose-induced transcriptional hysteresis occurs in glucose-sensing neurons over repetitive post-prandial experiences of elevated glucose. In turn, this phenomenon would be expected to enhance the sensitivity of these neurons to glucose signaling, thereby producing increased glycolysis in other parts of the body. Over a lifetime, this increased glycolysis would increase with age (which as discussed above does in fact occur), driving oxidative stress as described above. By the same token, DR would be expected to reduce the development of this metabolic profile (which again does in fact occur), thus reducing oxidative stress. While speculative, this hypothesis raises interesting new strategies for therapeutic interventions in diabetes and aging. The relevance of such responses to humans is demonstrated by such studies as those of Greco et al., who demonstrated that even 1 month after a modest reduction in caloric intake, obese humans demonstrated remarkable improvement of metabolic function, including enhanced insulin sensitivity and reduced plasma leptin (67).

## Role of CREB-Binding Protein in Mediating Toxic Effects of Glucose-Induced Transcriptional Hysteresis

Based on the hypothesis that glucose-induced transcriptional hysteresis drives the aging process and mediates the protective effects of DR, we used a high-throughput method to examine expression of hypothalamic genes regulating metabolism influenced by dietary restriction and hypoglycemia, which led to the studies described above (56, 57). However, a functional analysis based on the same data was also carried out, focusing on transcription factors regulated by fasting and hypoglycemia. From about 200 transcription factors putatively induced by fasting and/or hypoglycemia [such screens are notoriously prone to false positives (68)], we used two parallel high-throughput screens to discover glucose-regulated transcription factors most likely involved in regulating lifespan (69).

Carried out in parallel, we examined hypothalamic expression of transcription factors putatively induced by nutritional deprivation across five mouse strains with a range of average lifespans, while carrying out RNAi screens in *C. elegans* to discover transcription factors that would prevent life extension by DR (69). Remarkably, the same transcription factor was the top hit in both screens: CREB-binding protein (CBP). Hypothalamic expression of CBP predicted over 80% of variance in lifespan across the five strains of mice, and of all the transcription factors inhibited by RNAi, inhibition of CBP produced the most robust (indeed, complete) inhibition of the effect of DR to increase lifespan (69). CBP RNAi also prevented the protective effect of DR to delay pathology in a model of Alzheimer’s Disease (AD); indeed, CBP RNAi dramatically accelerated the pathology (69). Since CBP is a histone acetyltransferase, further studies demonstrated that the HDAC inhibitor, sodium butyrate, increases lifespan and delays pathology in the model of AD, dependent on CBP (69). Since there are different classes of DR that are dependent on different mechanisms (70), the role of CBP in the different classes was assessed, and all classes were dependent on CBP, making CBP uniquely required for protective effects of DR (69). Of particular importance to the role of glucose-induced transcriptional hysteresis, hypothalamic expression of CBP was inhibited both by diabetes and aging (69). These results suggest that glucose-induced transcriptional hysteresis to reduce hypothalamic expression of CBP could drive aspects of the aging process and that DR reduces this effect by reducing hypothalamic exposure to glucose. These results have been corroborated and extended by two independent highly qualified groups (71, 72). HDAC inhibitors have subsequently been shown to reverse pathology in mouse models of AD (73). Furthermore, it has now been shown that 3-OHB is an HDAC inhibitor (74), thus linking our studies with ketones and the ketogenic diet with the role of CBP in DR. Similarly, the reduction of impairments by DR in a mouse model of Huntington’s disease was associated with induction of hypothalamic CBP (75).

To assess the functional significance of hypothalamic CBP in mammals, CBP was ablated in adult mouse hypothalamus using a Cre-lox strategy (75). This led to rapid obesity and increased expression of CPT1a (75). Since CPT1a promotes FFA oxidation and FFAs are elevated during fasting, this observation supported the hypothesis that beta oxidation is an indicator of the fasting state to the hypothalamus. To test this hypothesis directly, CPT1a was overexpressed in the hypothalamus, which also caused obesity (75). This result raises the possibility that metabolism of lipids also exhibits a form of transcriptional hysteresis similar to that produced by glucose, possibly mediated by a member of the PPAR family of transcriptional factors. This hypothesis is currently being examined.

In conclusion, these studies indicate that exposure to high levels of glucose over a relatively short period, or even normal levels of glucose over the lifespan, lead to persistent elevation of glycolysis. In turn, glycolysis could drive obesity and diabetes, by driving persistent elevation of insulin secretion leading to obesity and pancreatic burn-out. Similarly, persistently elevated glycolysis could drive diabetic complications and aging by increasing oxidative stress. However, it may be possible to reverse these effects by a ketogenic diet, which blocks and apparently even reverses molecular effects of glucose.

## Ethics Statement

All animal studies were approved by the Mount Sinai IACUC.

## Author Contributions

The author (CM) conceived, designed, and supervised all aspects of the studies from his laboratory. He wrote the review and takes full responsibility for all aspects of the review and the data from his laboratory.

## Conflict of Interest Statement

The author declares that the research was conducted in the absence of any commercial or financial relationships that could be construed as a potential conflict of interest. The reviewer GM and handling Editor declared their shared affiliation.
